# Supplementary material for: Outpatient Nephrotoxic Medication Prescription after Pediatric Intensive Care Acute Kidney Injury
Source: Children (Basel). 2021 Oct 21;8(11):948. doi: 10.3390/children8110948 (PMC8618325; doi:10.3390/children8110948)
Supplement: Supplementary file 1 [file children-08-00948-s001.zip › Table S1.pdf]

Table S1. List of Nephrotoxic Medications Included in the Study with RAMQ (provincial drug plan coverage) Code, Drug Name, Pharmacologic Category and Status as Established or Potential Nephrotoxic Medication

RAMQ code	Drug name	Pharmacologic Class	Established vs Potential
38561	amikacine (sulfate d')	Antibiotic, Aminoglycoside	E
4212	gentamicine (sulfate de)	Antibiotic, Aminoglycoside	E
44723	gentamicine (sulfate de)/ sodium (chlorure de)	Antibiotic, Aminoglycoside	E
46704	gentamycine	Antibiotic, Aminoglycoside	E
5122	kanamycine (sulfate de)	Antibiotic, Aminoglycoside	E
6409	néomycine (sulfate de)	Antibiotic, Aminoglycoside	E
17828	néomycine (sulfate de)	Antibiotic, Aminoglycoside	E
6422	néomycine (sulfate de)/ gramicidine	Antibiotic, Aminoglycoside	E
43046	nétilmicine (sulfate de)	Antibiotic, Aminoglycoside	E
9139	streptomycine (sulfate de)	Antibiotic, Aminoglycoside	E
42877	tobramycine	Antibiotic, Aminoglycoside	E
33816	tobramycine (sulfate de)	Antibiotic, Aminoglycoside	E
46560	tobramycine (sulfate de)	Antibiotic, Aminoglycoside	E
2145	colistiméthate sodique	Antibiotic, Miscellaneous	E
42981	pipéracilline sodique	Antibiotic, penicillin	E
47097	pipéracilline sodique/ tazobactam sodique	Antibiotic, penicillin	E
43085	triméthoprime/ sulfaméthoxazole	Antibiotic, Sulfonamide Derivative	E
520	amphotéricine b	Antifungal Agent	E
46405	amphotéricine B	Antifungal Agent	E
5330	lithium (carbonate de)	Antimanic Agent	E
47237	lithium (carbonate de)	Antimanic Agent	E
47071	lithium (citrate de)	Antimanic Agent	E
40966	cisplatine	Antineoplastic Agent, Alkylating Agent	E

45508	ifosfamide	Antineoplastic Agent, Alkylating Agent	E
47651	méthotrexate	Antineoplastic Agent, Antimetabolite (Antifolate)	E
47167	indinavir (sulfate d')	Antiretroviral, Protease Inhibitor	E
46314	indinavir (sulfate d')	Antiretroviral, Protease Inhibitor	E
44099	acyclovir	Antiviral Agent	E
44034	acyclovir sodique	Antiviral Agent	E
47187	valacyclovir (chlorhydrate de)	Antiviral agent	E
47435	valganciclovir (chlorhydrate de)	Antiviral agent	E
46824	valganciclovir (chlorhydrate de)	Antiviral agent	E
47134	famciclovir	Antiviral Agent	E
46280	famciclovir	Antiviral Agent	E
47143	ganciclovir	Antiviral agent	E
46652	ganciclovir	Antiviral agent	E
45550	ganciclovir sodique	Antiviral agent	E
45628	pamidronate disodique	Bisphosphonate Derivative	E
44060	cyclosporine	Calcineurin Inhibitor	E
46329	cyclosporine	Calcineurin Inhibitor	E
46375	cyclosporine	Calcineurin Inhibitor	E
47152	tacrolimus	Calcineurin Inhibitor	E
46483	tacrolimus	Calcineurin Inhibitor	E
10036	vancomycine (chlorhydrate de)	Glycopeptide	E
47327	célécoxib	NSAID	E
46546	celecoxib	NSAID	E
47078	diclofénac (potassique ou sodique)	NSAID	E
47768	diclofénac diéthylamine	NSAID	E
41694	diclofénac sodique	NSAID	E
47059	diclofénac sodique/misoprostol	NSAID	E
43150	diflunisal	NSAID	E
47122	étodolac	NSAID	E
46256	étodolac	NSAID	E
33803	fenoprofène calcique	NSAID	E
44749	flurbiprofène	NSAID	E
4745	ibuprofène	NSAID	E

46654	ibuprofène	NSAID	E
47570	ibuprofène/ méthocarbamol	NSAID	E
47506	ibuprofène/ pseudoéphédrine (chlorhydrate de)	NSAID	E
4810	indométhacine	NSAID	E
38691	kétoprofène	NSAID	E
47066	kétorolac (trométhamine de)	NSAID	E
46006	kétorolac (trométhamine de)	NSAID	E
46150	ketorolac trométhamine	NSAID	E
47631	lumiracoxib	NSAID	E
44359	méfénamique (acide)	NSAID	E
47385	méloxicam	NSAID	E
47084	nabumétone	NSAID	E
46228	nabumetone	NSAID	E
46626	naproxen	NSAID	E
46152	naproxen sodique	NSAID	E
46335	naproxen sodique	NSAID	E
19752	naproxène	NSAID	E
47890	naproxène/ésoméprazole	NSAID	E
7462	phénylbutazone	NSAID	E
42019	piroxicam	NSAID	E
46638	piroxicam-B-cyclodextrine	NSAID	E
40381	sulindac	NSAID	E
45592	tenoxicam	NSAID	E
45407	tiaprofénique (acide)	NSAID	E
37664	tolmétine sodique	NSAID	E
46858	valdecoxib	NSAID	E
47140	lansoprazole	PPI	E
46283	lansoprozole	PPI	E
47850	dexlansoprazole	PPI	E
47418	esoméprazole (magnésium trihydraté)	PPI	E
46761	ésoméprazole magnésium	PPI	E
45519	oméprazole	PPI	E
47622	oméprazole (base ou magnésien)	PPI	E
47146	oméprazole magnésien	PPI	E
46713	oméprazole magnésien	PPI	E
47616	pantoprazole (magnésien ou sodique)	PPI	E

47234	pantoprazole sodique	PPI	E
46365	pantoprazole sodique	PPI	E
47432	rabéprazole sodique	PPI	E
47107	salsalate	Salicylate	E
46208	salsalate	Salicylate	E
45308	5-aminosalicylique (acide)	Salicylate	E
143	acétylsalicylique (acide)	Salicylate	E
46353	acétylsalicylique (acide)	Salicylate	E
46372	acetylsalicylique (acide)/ caféine/ codéine (phosphate)	Salicylate	E
46232	acétylsalicylique (acide)/ calcium (carbonate de)/ magnésium (carbonate de)/ magnésium (oxyde de)	Salicylate	E
38184	acétylsalicylique (acide)/ codéine (phosphate de)	Salicylate	E
38132	acétylsalicylique (acide)/ dextropropoxyphène (napsylate de)	Salicylate	E
46344	acétylsalicylique (acide)/ oxycodone (chlorhydrate de)	Salicylate	E
46036	acétylsalicylique (acide)/ oxycodone(chlorhydrate d')	Salicylate	E
46172	acétylsalicylique (acide)/ phénobarbital/codéine (phosphate de)	Salicylate	E
46368	acétylsalicylique acide/ caféine/codéine (phosphate)/ méprobamate	Salicylate	E
46077	acetylsalicylique acide/ dipyridamole	Salicylate	E
403	aminosalicylate de sodium	Salicylate	E
46583	bismuth (subsalicylate de)	Salicylate	E
46133	choline (salicylate de)/ (choline salicylate)	Salicylate	E
47365	dipyridamole/ acétylsalicylique (acide)	Salicylate	E
45517	ethylhexyl-P-methoxycinnate/ oxybenzone/ 2-ethylhexyl 2-cyano-3, 3-diphenyl acrylate/ 2-ethylhexyl salicylate	Salicylate	E
46128	methocarbamol/ acétylsalicylique (acide)	Salicylate	E
46098	méthocarbamol/ acétylsalicylique (acide)/ codéine phosphate)	Salicylate	E
47751	nifédipine/acétylsalicylique (acide)	Salicylate	E
46094	orphénadrine (citrate de)/ acétylsalicylique (acide)/ caféine	Salicylate	E
13091	physostigmine (salicylate de)	Salicylate	E

47595	pravastatine sodique/ acétylsalicylique (acide)	Salicylate	E
46198	propoxyphène (napsylate)/ acétylsalicylique (acide)/ caféine	Salicylate	E
8788	salicylazosulfapyridine	Salicylate	E
8801	salicylique (acide)	Salicylate	E
13468	salicylique (acide)	Salicylate	E
47127	iodixanol	Iodinated Contrast Media	P
45100	iohexol	Iodinated Contrast Media	P
45074	iopamidol	Iodinated Contrast Media	P
45600	ioversol	Iodinated Contrast Media	P
47583	gadobénate de diméglumine	Gadolinium-Containing Contrast Agent	P
47495	gadobutrol	Gadolinium-Containing Contrast Agent	P
47114	gadodiamide	Gadolinium-Containing Contrast Agent	P
47734	gadofosvéset trisodique	Gadolinium-Containing Contrast Agent	P
47108	gadopentétate de diméglumine	Gadolinium-Containing Contrast Agent	P
47011	gadotéridol	Gadolinium-Containing Contrast Agent	P
38834	rifampine	Antibiotic, Miscellaneous	P
47244	rifampine/ isoniazide/ pyrazinamide	Antibiotic, Miscellaneous	P
45403	carboplatine	Antineoplastic Agent, Alkylating Agent	P
47049	bénazépril (chlorhydrate de)	ACE inhibitor	P
42071	captopril	ACE inhibitor	P
47056	cilazapril	ACE inhibitor	P
47320	cilazapril/ hydrochlorothiazide	ACE inhibitor	P
45476	énalapril (maléate d')	ACE inhibitor	P
45572	énalapril (maléate d')/ hydrochlorothiazide	ACE inhibitor	P
47025	énalaprinate	ACE inhibitor	P
47002	fosinopril sodique	ACE inhibitor	P
45576	lisinopril	ACE inhibitor	P
47040	lisinopril/ hydrochlorothiazide	ACE inhibitor	P
47117	perindopril erbumine	ACE inhibitor	P
46258	perindopril erbumine	ACE inhibitor	P

47449	périndopril erbumine/indapamide	ACE inhibitor	P
45629	quinapril (chlorhydrate de)	ACE inhibitor	P
47301	quinapril (chlorhydrate de) hydrochlorothiazide	ACE inhibitor	P
47079	ramipril	ACE inhibitor	P
46216	ramipril	ACE inhibitor	P
47655	ramipril/ hydrochlorothiazide	ACE inhibitor	P
47250	trandolapril	ACE inhibitor	P
47440	trandolapril/ vérapamil (chlorhydrate de)	ACE inhibitor	P
41551	céfadroxil (monohydrate de)	Antibiotic, Cephalosporin (First Generation)	P
45422	céfadroxil anhydre	Antibiotic, Cephalosporin (First Generation)	P
18725	céfazoline sodique	Antibiotic, Cephalosporin (First Generation)	P
1612	céphalexine (monohydrate de)	Antibiotic, Cephalosporin (First Generation)	P
1638	céphalotine sodique	Antibiotic, Cephalosporin (First Generation)	P
19375	céphapirine sodique	Antibiotic, Cephalosporin (First Generation)	P
19739	céphradine	Antibiotic, Cephalosporin (First Generation)	P
47149	céfépime (chlorhydrate de)	Antibiotic, Cephalosporin (Fourth Generation)	P
42032	céfaclor	Antibiotic, Cephalosporin (Second Generation)	P
39601	céfamandole (nafate de)	Antibiotic, Cephalosporin (Second Generation)	P
45559	céfotétane disodique	Antibiotic, Cephalosporin (Second Generation)	P
44827	céfoxitine sodique	Antibiotic, Cephalosporin (Second Generation)	P
40433	céfoxitine sodique	Antibiotic, Cephalosporin (Second Generation)	P
47139	cefprozil	Antibiotic, Cephalosporin (Second Generation)	P
45573	céfuroxime axetil	Antibiotic, Cephalosporin (Second Generation)	P
41616	céfuroxime sodique	Antibiotic, Cephalosporin (Second Generation)	P
45546	cefixime	Antibiotic, Cephalosporin (Third Generation)	P

44450	céfopérazone sodique	Antibiotic, Cephalosporin (Third Generation)	P
42799	céfotaxime sodique	Antibiotic, Cephalosporin (Third Generation)	P
45615	ceftazidime	Antibiotic, Cephalosporin (Third Generation)	P
45152	ceftazidime (pentahydrate de)	Antibiotic, Cephalosporin (Third Generation)	P
45548	ceftizoxime sodique	Antibiotic, Cephalosporin (Third Generation)	P
45473	ceftriaxone disodique	Antibiotic, Cephalosporin (Third Generation)	P
47669	ceftriaxone sodique	Antibiotic, Cephalosporin (Third Generation)	P
45503	ciprofloxacine (chlorhydrate de)	Antibiotic, Fluoroquinolone	P
45619	ciprofloxacine	Antibiotic, Fluoroquinolone	P
47258	lévofloxacine	Antibiotic, Fluoroquinolone	P
47391	moxifloxacine (chlorhydrate de)	Antibiotic, Fluoroquinolone	P
46698	moxifloxacine(chlorhydrate de)	Antibiotic, Fluoroquinolone	P
45443	norfloxacine	Antibiotic, Fluoroquinolone	P
45604	ofloxacine	Antibiotic, Fluoroquinolone	P
6331	nalidixique (acide)	Antibiotic, Fluoroquinolone	P
2431	dapsone	Antibiotic, Miscellaneous	P
18244	amoxicilline	Antibiotic, penicillin	P
44333	amoxicilline/ clavulanate de potassium	Antibiotic, penicillin	P
46515	amoxicilline/clavulanate de potassium	Antibiotic, penicillin	P
533	ampicilline	Antibiotic, penicillin	P
546	ampicilline sodique	Antibiotic, penicillin	P
18257	ampicilline/ probénécide	Antibiotic, penicillin	P
45487	bacampicilline (chlorhydrate de)	Antibiotic, penicillin	P
47292	lansoprazole/ amoxicilline/ clarithromycine	Antibiotic, penicillin	P
6318	nafcilline sodique	Antibiotic, Penicillin	P
6773	oxacilline sodique	Antibiotic, Penicillin	P

42110	pénicilline g (ammoniacale ou potassique)	Antibiotic, penicillin	P
7007	pénicilline g (benzathine)	Antibiotic, penicillin	P
7020	pénicilline g (benzathine)/ pénicilline g procaïnique/ pénicilline g potassique	Antibiotic, penicillin	P
7033	pénicilline g ammoniacale	Antibiotic, penicillin	P
7046	pénicilline g potassique	Antibiotic, penicillin	P
7059	pénicilline g procaïnique	Antibiotic, penicillin	P
7072	pénicilline G sodique	Antibiotic, penicillin	P
7371	phénoxyméthylpénicilline	Antibiotic, penicillin	P
42123	phénoxyméthylpénicilline (base ou sel potassique)	Antibiotic, penicillin	P
7384	phénoxyméthylpénicilline (benzathine)	Antibiotic, penicillin	P
7397	phénoxyméthylpénicilline potassique	Antibiotic, penicillin	P
45424	pivampicilline	Antibiotic, penicillin	P
45191	pivmécilliname (chlorhydrate de)	Antibiotic, penicillin	P
38743	ticarcilline disodique	Antibiotic, penicillin	P
45560	ticarcilline disodique/ clavulanate de potassium	Antibiotic, penicillin	P
47229	topiramate	Anticonvulsant, Miscellaneous	P
46359	topiramate	Anticonvulsant, Miscellaneous	P
45557	pentamidine (iséthionate de)	Antifungal Agent, Antiprotozoal	P
45507	pentamidine iséthionate	Antifungal Agent, Antiprotozoal	P
4524	hydralazine (chlorhydrate d')	Antihypertensive; Vasodilator	P
8489	quinine (sulfate de)	Antimalarial Agent	P
47307	clopidogrel (bisulfate de)	Antiplatelet Agent, Thienopyridine	P
46486	clopidogrel	Antiplatelet Agent, Thienopyridine	P
47397	lopinavir/ ritonavir	Antiretroviral, Protease Inhibitor	P
46714	lopinavir/ritonavir	Antiretroviral, Protease Inhibitor	P
47170	ritonavir	Antiretroviral, Protease Inhibitor	P
46313	ritonavir	Antiretroviral, Protease Inhibitor	P

47479	atazanavir (sulfate d')	Antiretroviral, Protease Inhibitor	P
8242	propylthiouracile	Antithyroid agent, thioamide	P
47877	boceprevir, ribavirine, peginterféron alfa 2B	Biological Response Modulator	P
47886	bocéprévir/ribavirine/interféron alfa-2B péguylé	Biological Response Modulator	P
45444	interféron alfa-2b	Biological Response Modulator	P
47314	interféron alfa-2b (sans albumine humaine)	Biological Response Modulator	P
47406	interféron alfa-2B péguylé	Biological Response Modulator	P
47321	ribavirine/ interferon alfa-2B	Biological Response Modulator	P
47445	ribavirine/ interféron alfa-2B péguylé	Biological Response Modulator	P
46849	ribavirine/interféron alfa- 2B pégylé	Biological Response Modulator	P
46535	ribavirine/interferon alfa-2B	Biological Response Modulator	P
47386	zolédronique (acide)	Bisphosphonate Derivative	P
46695	zolidronique acide	Bisphosphonate Derivative	P
45256	auranofine	Gold Compound	P
715	aurothiomalate de sodium	Gold Compound	P
43163	ranitidine (chlorhydrate de)	Histamine H2 Antagonist	P
47257	ranitidine citrate de bismuth	Histamine H2 Antagonist	P
47573	bevacizumab	Monoclonal Antibody, VEGF inhibitor	P
47800	temsirolimus	mTOR Kinase Inhibitor	P
47411	sirolimus	mTOR Kinase Inhibitor	P
46736	sirolimus	mTOR Kinase Inhibitor	P
47607	adéfovir dipivoxil	Reverse Transcriptase Inhibitor	P
47724	éfavirenz/ emtricitabine/ ténofovir disoproxil (fumarate de)	Reverse Transcriptase Inhibitor	P
47605	emtricitabine/ ténofovir disoproxil (fumarate de)	Reverse Transcriptase Inhibitor	P
47502	tenofovir disoproxil	Reverse Transcriptase Inhibitor	P
47512	ténofovir disoproxil (fumarate de)	Reverse Transcriptase Inhibitor	P

47324	abacavir (sulfate d')	Reverse Transcriptase Inhibitor, Nucleoside	P
47422	abacavir (sulfate d') / lamivudine / zidovudine	Reverse Transcriptase Inhibitor, Nucleoside	P
46564	abacavir (sulfate d')	Reverse Transcriptase Inhibitor, Nucleoside	P
47576	abacavir (sulfate d')/lamivudine	Reverse Transcriptase Inhibitor, Nucleoside	P
46769	abacavir sulfate/lamivudine/ zidovudine	Reverse Transcriptase Inhibitor, Nucleoside	P
45611	didanosine	Reverse Transcriptase Inhibitor, Nucleoside	P
46581	didanosine	Reverse Transcriptase Inhibitor, Nucleoside	P
47147	lamivudine	Reverse Transcriptase Inhibitor, Nucleoside	P
46514	lamivudine	Reverse Transcriptase Inhibitor, Nucleoside	P
47310	lamivudine/ zidovudine	Reverse Transcriptase Inhibitor, Nucleoside	P
46520	lamivudine/zidovudine	Reverse Transcriptase Inhibitor, Nucleoside	P
195	allopurinol	Xanthine Oxidase Inhibitor	P
