# Supplementary material for: Outpatient Nephrotoxic Medication Prescription after Pediatric Intensive Care Acute Kidney Injury
Source: Children (Basel). 2021 Oct 21;8(11):948. doi: 10.3390/children8110948 (PMC8618325; doi:10.3390/children8110948)
Supplement: Supplementary file 1 [file children-08-00948-s001.zip › Table S2.pdf]

Table S2. Comparisons between included and excluded children

Characteristics	Included<br>N (%)	Excluded; did not have ≥30 days RAMQ coverage<br>N (%)	
N (denominator)	970	1421	
Baseline Factors			
Age, years<br>Median [IQR]	4 [1-11]	4 [1-11]	0.37
Female Sex (%)	423 (44)	632 (45)	0.71
Prior CKD	48 (5)	76 (5)	0.71
4th/5th quintile social deprivation index	476 (52)	442 (33)	<0.0001
4th/5th quintile material deprivation index	529 (57)	458 (34)	<0.0001
Rural home address	148 (15)	227 (16)	0.57
PICU Diagnosis			
Cardiac surgery	141 (15)	209 (15)	0.95
Cardiac (non-surgical)	42 (4)	90 (6)	0.04
Trauma	82 (8)	141 (10)	0.25
Renal	5 (0.5)	14 (1)	0.25
Infection (non-bronchiolitis)	163 (170	235 (17)	0.87
Neurologic/neurosurgical	117 (12)	181 (13)	0.66
Gastrointestinal	34 (4)	43 (3)	0.56
Oncologic	20 (2)	38 (3)	0.42
Respiratory	91 (9)	135 (10)	0.94
Diabetes	26 (3)	33 (2)	0.59
Other admission diagnosis	249 (26)	302 (21)	0.01
PICU Variables			
Pediatric medical complexity<br>Non-chronic<br>Non-complex chronic<br>Complex chronic	 139 (14)<br>223 (23)<br>608 (63)	 260 (18)<br>302 (21)<br>859 (60)	 0.05
Postoperative (non-cardiac)	141 (15)	209 (15)	0.95
Vasopressors used	174 (18)	241(17)	0.55
Diuretics used	258 (27)	384 (27)	0.85
Steroids used	269 (28)	355 (25)	0.14
Antibiotics used	683 (70)	979 (69)	0.44
PRISM score<br>Median [IQR]	7 [4-11]	7 [4-11]	0.78
Mechanically ventilated	452 (47)	638 (45)	0.43
PICU length of stay, days<br>Median [IQR]	1 [1-4]	2 [1-4]	0.91
Hospital length of stay, days<br>Median [IQR]	9 [5-18]	8 [5-16]	0.33

Renal Factors in PICU			
Renal diagnosis on admission	5 (1)	14 (1)	0.25
Renal replacement therapy	5 (1)	7 (0)	1.00
Nephrology consult	58 (6)	97 (7)	0.45
Nephrotoxic antibiotics	193 (20)	312 (22)	0.24
NSAIDs	124(13)	157 (11)	0.20
Aminoglycosides	115 (12)	184 (13)	0.45
Acyclovir/ganciclovir	27 (3)	47 (3)	0.55
Amphotericin	4 (0)	13 (1)	0.22
Vancomycin	100 (10)	149 (10)	0.95
