# Supplementary material for: Outpatient Nephrotoxic Medication Prescription after Pediatric Intensive Care Acute Kidney Injury
Source: Children (Basel). 2021 Oct 21;8(11):948. doi: 10.3390/children8110948 (PMC8618325; doi:10.3390/children8110948)
Supplement: Supplementary file 1 [file children-08-00948-s001.zip › Table S3.pdf]

Table S3. Patient characteristics by AKI status and by outcome status during the whole follow-up

Characteristics	AKI	No AKI
N (denominator)	223	747
Baseline Factors		
Age, years (age)<br>Median [IQR]	3.62<br>[0.61, 10.14]	3.68<br>[1.04, 11.08]
Female Sex (%)	99 (44.4%)	324 (43.4%)
Prior CKD	6 (2.7%)	17 (2.3%)
$4^{th}/5^{th}$ quintile social deprivation index[a]	105 (50.2%)	371 (51.9%)
$4^{th}/5^{th}$ quintile material deprivation index[a]	116 (55.5%)	413 (57.8%)
Rural home address[b]	31 (14.4%)	117 (15.8%)
PICU Diagnosis		
Cardiac surgery	75 (33.6%)	66 (8.8%)*
Cardiac (non-surgical)	10 (4.5%)	32 (4.3%)
Trauma	15 (6.7%)	67 (9.0%)
Renal	4 (1.8%)	1 (0.1%)*
Infection (non-bronchiolitis)	34 (15.2%)	129 (17.3%)
Neurologic/neurosurgical	17 (7.6%)	100 (13.4%)*
Gastrointestinal	13 (5.8%)	21 (2.8%)
Oncologic	3 (1.3%)	17 (2.3%)
Respiratory	14 (6.3%)	77 (10.3%)
Diabetes	10 (4.5%)	16 (2.1%)
Other admission diagnosis	28 (12.6%)	221 (29.6%)*
PICU Variables		
Pediatric medical complexity<br>Non-chronic<br>Non-complex chronic<br>Complex chronic	 18 (8.1%)<br>33 (14.8%)<br>172 (77.1%)	 121 (16.2%)*<br>190 (25.4%)*<br>436 (58.4%)*
Postoperative (non-cardiac)	45 (20.2%)	252 (33.7%)*
Vasopressors used	95 (42.6%)	79 (10.6%)*
Diuretics used	121 (54.3%)	137 (18.4%)*
Steroids used	72 (32.3%)	197 (26.4%)
Antibiotics used	183 (82.1%)	500 (66.9%)*
PRISM score<br>Median [IQR]	11.00<br>[6.00, 16.50]	6.00<br>[4.00, 10.00]*
Mechanically ventilated	155 (69.5%)	297 (39.8%)*
PICU length of stay, days<br>Median [IQR]	3.53<br>[1.68, 8.07]	1.07<br>[0.76, 2.58]*
Hospital length of stay, days<br>Median [IQR]	14.00<br>[8.00, 29.00]	8.00<br>[4.00, 14.00]*
Renal Factors in PICU		
Renal replacement therapy	5 (2.2%)	0 (0.0%)*
Nephrology consult	35 (15.7%)	23 (3.1%)*
Nephrotoxic antibiotics	70 (31.4%)	123 (16.5%)*

NSAIDs	42 (18.8%)	82 (11.0%)*
Aminoglycosides	48 (21.5%)	67 (9.0%)*
Acyclovir/ganciclovir	9 (4.0%)	18 (2.4%)
Amphotericin	2 (0.1%)	2 (0.3%)
Vancomycin	39 (17.5%)	61 (8.2%)*

Abbreviations: AKI: acute kidney injury; IQR: interquartile range; CKD: chronic kidney disease; PICU: pediatric intensive care unit; PRISM: pediatric risk of mortality; NSAID: non-steroidal anti-inflammatory drug

[a] Total number of patients was lower for these variables (denominators by group: n=209 for AKI; n=715 for non-AKI; n=399 for <3 nephrotoxic medications during follow-up; n=525 for ≥3 nephrotoxic medications during follow-up).

[b] Total number of patients was lower for these variables (denominators by group: n=215 for AKI; n=741 for non-AKI; n=415 for <3 nephrotoxic medications during follow-up; n=541 for ≥3 nephrotoxic medications during follow-up)

*p-value<0.05
