# Supplementary material for: Outpatient Nephrotoxic Medication Prescription after Pediatric Intensive Care Acute Kidney Injury
Source: Children (Basel). 2021 Oct 21;8(11):948. doi: 10.3390/children8110948 (PMC8618325; doi:10.3390/children8110948)
Supplement: Supplementary file 1 [file children-08-00948-s001.zip › Table S4.pdf]

Table S4. Adjusted Association Between AKI and Receiving ≥3 Potential Nephrotoxic Medication Prescriptions and Classes

	**AKI *vs.* no AKI**		**Stage 2/3 *vs.* no AKI or Stage 1 AKI**	
	**First year (n=953) Adjusted RR (95% CI)**	**First 5 years (n=910) Adjusted RR (95% CI)**	**First year (n=953) Adjusted RR (95% CI)**	**First 5 years (n=910) Adjusted RR (95% CI)**
**≥3 potential nephrotoxic medication prescriptions**	1.01 (0.94-1.09)[a]	1.06 (0.88-1.28)[b]	1.03 (0.93-1.15)[a]	1.17 (0.87-1.59)[b]
**≥3 potential nephrotoxic medication *classes***	1.03 (0.97-1.09)[c]	1.07 (1.00-1.16)[d]	1.03 (0.94-1.12)[c]	1.10 (0.97-1.25)[d]

Abbreviations: AKI: Acute Kidney Injury; RR: Risk Ratio; ACE-inhibitors: Angiotensin Converting Enzyme inhibitors

NB: All models include covariates: age, gender, rural versus urban home address, pediatric medical complexity algorithm and social and material deprivation indices. Additional model covariates (associated with the exposure and outcome of the analysis) include:

a. cardiac surgery, trauma, diabetes, diuretics, antibiotics, PRISM score
b. cardiac surgery, trauma, diabetes, diuretics, antibiotics
c. cardiac surgery, trauma, diuretics, antibiotics
