# Supplementary material for: Outpatient Nephrotoxic Medication Prescription after Pediatric Intensive Care Acute Kidney Injury
Source: Children (Basel). 2021 Oct 21;8(11):948. doi: 10.3390/children8110948 (PMC8618325; doi:10.3390/children8110948)
Supplement: Supplementary file 1 [file children-08-00948-s001.zip › Table S5.pdf]

Table S5. Dispensed medications in children with AKI

Medication Class	Frequency	Percent	Cumulative Frequency	Cumulative Percent
**BETA-LACTAMS**	1064	22.55	1064	22.55
**PPI**	683	14.47	1747	37.02
**ACE-INHIBITOR**	575	12.18	2322	49.21
**OTHER ANTIBIOTIC**	509	10.79	2831	59.99
**SALICYLATE**	479	10.15	3310	70.14
**IMMUNOMODULATOR**	469	9.94	3779	80.08
**HIV MEDICATIO**	371	7.86	4150	87.94
**OTHER**	292	6.19	4442	94.13
**NSAID**	141	2.99	4583	97.12
**ANTIVIRAL**	72	1.53	4655	98.64
**AMINOGLYCOSIDE**	64	1.36	4719	100.00

Abbreviations: PPI: proton-pump inhibitors; ACE: angiotensin-converting enzyme; HIV: human immunodeficiency virus; NSAID: non-steroidal anti-inflammatory drug
