# Supplementary material for: Outpatient Nephrotoxic Medication Prescription after Pediatric Intensive Care Acute Kidney Injury
Source: Children (Basel). 2021 Oct 21;8(11):948. doi: 10.3390/children8110948 (PMC8618325; doi:10.3390/children8110948)
Supplement: Supplementary file 1 [file children-08-00948-s001.zip › Table S6.pdf]

Table S6. Dispensed medications for children with no AKI

Medication Class	Frequency	Percent	Cumulative Frequency	Cumulative Percent
**BETA-LACTAMS**	3218	41.85	3218	41.85
**PPI**	1006	13.08	4224	54.94
**OTHER ANTIBIOTIC**	834	10.85	5058	65.78
**OTHER**	829	10.78	5887	76.56
**ACE-INHIBITOR**	480	6.24	6367	82.81
**AMINOGLYCOSIDE**	470	6.11	6837	88.92
**SALICYLATE**	403	5.24	7240	94.16
**NSAID**	230	2.99	7470	97.15
**HIV MEDICATION**	100	1.30	7570	98.45
**IMMUNOMODULATOR**	70	0.91	7640	99.36
**ANTIVIRAL AGE**	46	0.60	7686	99.96
**ANTIFUNGAL**	3	0.04	7689	100.00

Abbreviations: PPI: proton-pump inhibitors; ACE: angiotensin-converting enzyme; NSAID: non-steroidal anti-inflammatory drug; HIV: human immunodeficiency virus
